# Intended Pregnancy as a Predictor of Good Knowledge on Birth Preparedness and Complication Readiness: the Case of Northern Ethiopia Pregnant Mothers

**DOI:** 10.1155/2019/9653526

**Published:** 2019-01-21

**Authors:** Haile Zewdu Tsegaw, Endeshaw Admassu Cherkos, Marta Berta Badi, Muhabaw Shumye Mihret

**Affiliations:** ^1^Maternity Ward, Gondar University Comprehensive and Specialized Referal Hospital, Ethiopia; ^2^Department of Midwifery, College of Medicine and Health Sciences, University of Gondar, Ethiopia

## Abstract

**Background:**

Maternal mortality remains unacceptably high in developing countries. One key strategy to reduce such mortality is utilization of birth preparedness and complication readiness (BP/CR) and creating awareness of BP/CR is an important step for pregnant women, their families, and the community. However, there was limited to no evidence regarding the community's awareness on BP/CR in the study area. Therefore, this study aimed to assess knowledge on BP/CR and associated factors among pregnant women in Debremarkos town, Northwest Ethiopia, 2017.

**Methods:**

A Community based cross-sectional study was conducted from July 1 to 30/2017. A total of 441 pregnant women were included in the study. Structured and pretested questionnaire was administered through face to face interview to collect the data. Simple random sampling technique was used to select the study participants. The data were entered in to Epinfo version 7.0 and then exported to SPSS version 20.0 for analysis. Both bivariate and multivariable logistic regression model were fitted. Crude and adjusted odds ratio with 95 % confidence interval have been computed and variables with p-value < 0.05 were considered statistically significance*. Results*. The proportion of pregnant women having good knowledge on birth preparedness and complication readiness was found to be 45.2 with 95%CI (40.4, 50.0). In the multivariable analysis, having history of childbirth (AOR=2.17;95%CI:1.18,4.00), having intended pregnancy (AOR=2.13;95%CI: 1.16, 3.90), being governmental employee ( AOR=6.50; 95%CI: 2.50, 16.87), and having Antenatal care visits (AOR=5.50; 95%CI:2.2,13.70) were factors which were independently and significantly associated with good knowledge on birth preparedness and complication readiness.

**Conclusion:**

Proportion of pregnant women having good knowledge on birth preparedness and complication readiness was low. Putting emphasis on intended pregnancy and antenatal care visit was recommended.

## 1. Introduction

Reduction of maternal mortality has been continued to be a global top public health identified problem [1]. About 830 women daily and 303,000 women annually die from pregnancy and/or childbirth-related complications globally by the year 2015. Almost all of these deaths occurred in low-resource settings, and most could have been prevented [2, 3]. It remains unacceptably high in least developed regions especially in West & Central Africa (WCA) and Sub-Saharan Africa (SSA). According to the United Nations Children's Fund (UNICEF) 2017 report, maternal mortality ratio (MMR) was 216 in globe, 679 in WCA, and 546 in SSA per 100, 000 live births [4]. As a SSA country, Ethiopia has also been suffered from similar problem [5–7]. According to Ethiopian Demographic Health Survey (EDHS) 2016 report, MMR in Ethiopia was 412/100,000 live births [8].

To reduce high maternal mortality, World Health Organization (WHO) in cooperation with its partners has been designing various strategies and standards [9, 10]. From the strategies, birth preparedness and complication readiness (BP/CR) is among the key strategies which have been intended to promote the timely use of skilled maternal and neonatal care [11, 12]. BP/CR is the process of planning for normal birth and anticipating the actions needed in case of an emergency [13]. Moreover, it encourages the pregnant women, the families, and the community at large to have an active preparation to prevent delay in receiving basic emergency obstetric and neonatal care (BEmONC) [14]. Every pregnant woman may face the risk of unpredictable and sudden complications that could end in death or injury [15]. Hence, pregnant women, their family, and the community need to plan for the care needed during the antepartum, intrapartum, and postpartum period and prepare themselves to take action in emergencies [16].

The main components of the BP/CR include recognizing danger signs, identifying skilled birth attendant, saving money, identifying mode of transport, identifying where to go in case of complications, and identifying a blood donor [17]. However, lack of advanced planning for use of skilled birth attendant for normal birth [18] and particularly inadequate preparation for rapid action in the event of obstetric complication are well documented factors contributing to delay in receiving skilled obstetric care [19–21]. Despite its significance, large number of pregnant women and their families may not be aware of the components of BP/CR which in turn may bring about low practice of BP/CR.

Despite the great potential of BP/CR in reducing the maternal and newborn deaths, there is a dearth of evidence on what proportions of women are aware of BP/CR in the study area. Moreover, it is crucial to identify the factors contributing for the woman's knowledge on BP/CR within the community context to design effective BP/CR awareness strategies. In doing so, this study was aimed to assess knowledge on BP/CR and its associated factors among pregnant women living in Debremarkos town.

## 2. Methods

A community based cross-sectional study was conducted from July 1 to 30/2017 in Debremarkos town, Northwest Ethiopia. The town which is located at 300 kilometers away from Addis Ababa-the capital city of Ethiopia is found at the Northwest direction and 265 kilometers away from Bahir Dar—the capital city of Amahara National regional state in the Southeast direction. It is also the administrative town of the East Gojam Zone which is among 11 Zones of Amhara national regional state. According to the Ethiopian population projection report for all regions at Woreda level conducted from 2014 to 2017, the total population of the town was estimated to be 92,470. Among these, about 45,732 were males and 46,738 were females [22]. From these, women in the reproductive age group were 14,618 and the numbers of households in the town were estimated to be 14,528.

In Ethiopia particularly in Debremarkos town, antenatal care (ANC) services are not active. In connection to this, health care providers have not gone to women's homes to provide formal ANC service even if the women do not attend the clinic. However, the health extension workers (HEWs) are anticipated to go home to home for awareness creation and community mobilization. Accordingly, HEWs are responsible to educate pregnant women, the family and even the community at large about birth preparedness and complication readiness; danger signs during pregnancy; childbirth and postpartum period; the importance of having ANC visit; when and how to get the skilled health care providers by moving home to home and/or arranging a discussion forum in the community in collaboration with the local governmental leaders and/or religious leaders. As far as duties for provision of ANC services are concerned, midwives are primarily responsible at the health centers and/or hospitals, whereas, health extension workers provide ANC services at health post (i.e., the smallest unit of health facility). Moreover, there is a consultancy and/or referral system across different health professionals and levels of health facilities. Accordingly, upon indications, HEWs at health posts refer cases to health centers and/or hospitals where midwives welcome and manage them. Depending on the type and severity of the problems, midwives may consult professionals who specialized in either Clinical Midwifery (Msc), Integrated Emergency Obstetrics and Surgery (Msc), or obstetrics and gynecology (doctors). In addition, midwives may refer eligible cases to higher level of health facilities as necessary.

All residents of Debrmarkos town's pregnant women (i.e., those pregnant women who have been living for at least six months in the town at the time of interview) were included in the study.

Single population proportion formula was used to calculate the sample size by assuming 50%, proportion of pregnant women having good knowledge on BP/CR; 95%, confidence interval; 5 %, margin of error; and 10%, nonresponse rate. Based on these assumptions, the total sample size was calculated using the following formula:

(1) $$\begin{array}{c} N=\frac{{Z_{\alpha /2}}^{2}.p\left(1\text{-}p\right)}{D^{2}}=\frac{{\left(1.96\right)}^{2}*\left(0.5\right)\left(0.5\right)}{{\left(0.05\right)}^{2}}=384 \end{array}$$

where N= required sample size, Z= confidence level at 95% (at standard value of 1.96), p= estimated proportion (50%), and d = margin of error at 5 % (standard value of 0.05). Upon substitution of the given values in the above formula, the calculated minimum sample size was found to be 384. By adding 15% nonresponse rate, the total sample size turned to be 441.

First, the list of total number of pregnant women was obtained from HEWs in Debremarkos town. Then, study participants were selected using simple random sampling technique using computer generating method. Afterward, the data collectors have employed a map that the town health extension workers have been using, to get the selected mothers. Moreover, the data collectors and supervisors have been guided by HEWs whenever they faced difficulties of getting the selected pregnant woman.

Sociodemographic and obstetric related variables such as age, marital status, religion, ethnicity, husband's educational status, husband's occupation, family monthly income, parity, gravidity, age at 1st marriage, age at first pregnancy, desired number of children, antenatal care (ANC) visit in the current pregnancy, and place of ANC visit in the current pregnancy and intended pregnancy were included. In addition, knowledge on BP/CR (‘Good' or ‘Poor') was assessed based on some criteria.

A selected pregnant woman was labeled as having good knowledge on BP/CR if she mentioned at least three out of the following five components of BP/CR: identified health facility for childbirth, saved money for birth, arranged transportation, arranged accompany, and arrange potential blood donor.

The data were collected using structured questionnaire through face to face interview. Six midwifery professionals were involved in data collection process. These included six unemployed midwives having diploma in midwifery as the data collectors and one midwife having Bsc in clinical midwifery as a supervisor. The questionnaire was adapted from previous similar research articles [19, 23]. It was first developed in English and then translated to local language (i.e., Amharic) and then back to English to facilitate the understanding of the respondents. A one-day training was provided for the data collectors and a supervisor regarding the objectives of the study, way of approach to the community, sampling techniques, sampling procedures, client privacy issue, client confidentiality issue, client informed and voluntary participation, data collection method, and significance of the study. Then after that, a pretest was conducted on 5% of sample size at Finoteselam, which is a nearby town for the study area, before the actual data collection started. The purpose and objectives of the study were clearly stated in the first page of the questionnaire which the interviewers have explained for participants. During data collection, there was a close communication among the data collectors, a supervisor, and a principal investigator. The collected questionnaires were checked for completeness and on spot corrective measures were taken both by data collectors and supervisors. Daily meeting has been conducted among the data collectors, a supervisor, and a principal investigator for discussion regarding presenting difficulties and to assess the progress of data collection.

Data were checked, coded, and entered into Epi-info7 then exported to SPSS version 20 software package for further analysis. Frequencies and cross tabulations were used to summarize descriptive statistics. The data were presented by texts, tables, and graphs. Furthermore, binary logistic regression was used to identify factors associated with knowledge on BP/CR. Thereafter, variables were fitted to multivariable logistic regression using backward likelyhood ratio method. Both crude odds ratio (COR) and adjusted odds ratio (AOR) with the corresponding 95%CI were computed to show the strength of the association. In addition, model fitness was checked using Hosmer and Lemeshow goodness of a fit test and the model test P-value was found to be 0.13. Finally, statistically significant association of variables was claimed based on AOR with its 95% CI and p-value <0.05.

## 3. Results

### 3.1. Sociodemographic Related Characteristics

A total of 436 pregnant women were participated in the study area. The participants' response rate was 98.9%. The mean age of respondents were 28.5 (SD ±6.8) and 67.7% of the women were within the age range of 18 -24 years. Majority (92.7%) of participants were Orthodox Tewahido Christian by religion. Moreover, three-fourth (77.5%) of respondents reported that they had an average family monthly income of more than 90 US Dollar. More than half (60%) of the male partners had attended education of college whereas 229 (52%) of male partners had been employed in governmental organizations [Table 1].

### 3.2. Obstetric Characteristics of the Respondents

About 200 (45.9%) and 198 (45.4%) of respondents were primiparous and nulliparous, respectively. Considerable number (6.9%) of respondents reported that they had gotten their first marriage at the age of <18 years. More than half (51.5%) of the participants' first pregnancies were within the age range of 18-24. Substantial (84.2%) of the current pregnancies were intended [Table 2].

### 3.3. Knowledge on Birth Preparedness and Complication Readiness

**Figure 1:**
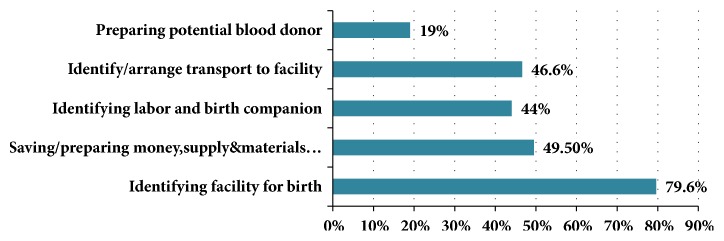
BP/CR elements mentioned by pregnant women in Debre Markos town, 2017.

The proportion of pregnant mothers having good knowledge on BP/CR was found to be 45.2% with 95%CI (40.4, 50.00). More than nine-tenth (92.2%) of mothers mentioned at least one element of BP/CR. Majority (79.6%) of pregnant women had identified the health facility where they planned to give birth at time of interview [Figure 1].

In addition, about 405 (92.9%) of the pregnant women previously had heard about BP/CR; of them 73.8% heard from health professionals, 15.8% from mass media, and 10.4% from relatives and/or friends.

### 3.4. Knowledge on Danger Signs of Antepartum, Intrapartum, Postpartum, and Newborn

Most (93.1%) of the respondents mentioned at least one key danger sign during pregnancy and 352 (80.7%) mentioned at least two danger signs of pregnancy. Three hundred ninety-nine (91.5%) of the respondents mentioned at least one and 326 (74.8%) of participants mentioned at least two danger signs during labor and childbirth. Three hundred seventy-one (85%) and 332 (76.2%) of pregnant women mentioned at least one and at least two danger signs during postpartum period, respectively, whereas about 347 (79.6%) and 239 (54.8%) of pregnant women mentioned at least one and at least two newborn danger signs, respectively [Table 3].

### 3.5. Factors Associated with Birth Preparedness and Complication Readiness

Based on the findings from bivariate logistic regression analysis, mother's educational status, mother's occupation, family monthly income, intended pregnancy, parity, and ANC visit have been associated with knowledge on BP/CR. However, in the multivariable logistic regression analysis, mother's occupation, intended pregnancy, parity, and ANC visit for the current pregnancy have remained statistically significantly associated with good knowledge on BP/CR.

Accordingly, the odds of having good knowledge on BP/CR were 6.50 times higher among governmental employed mothers than others (AOR=6.50; 95% CI: 2.50, 16.87). Likewise, mothers having current intended pregnancy were 2.1 times more likely to have good knowledge on BP/CR as compared with their congruent(AOR =2.10; 95% CI: 1.16, 3.90). Moreover, mothers having ANC visit in the current pregnancy were 5.50 times more likely to have good knowledge on BP/CR than those mothers who had no ANC visit (AOR =5.50, 95%CI; 2.20,13.70). Similarly, pregnant women with history of childbirth were 2.6 times (AOR =2.60, 95% CI; 1.18, 4.00) more likely to have good knowledge on BP/CR than a nulliparous pregnant women (Table 4).

## 4. Discussion

This community based cross-sectional study was conducted to assess knowledge towards BP/CR and associated factors among pregnant women living in Debremarkos town. The odds of good knowledge on BP/CR were higher among mothers who were governmentally employed, got the current pregnancy intentionally, had ANC follow-up during the current pregnancy and had history of previous childbirth.

In the current study, the proportion of mothers having good knowledge on BP/CR was 45.2%. This finding is comparable with the study conducted in Mpwapwa District Tanzania (43.1%) [24], Indore city India (47.8%) [25],Tewale teaching Hospital Ghana (43.7%) [26] and Morang district Nepal (45.2%) [27]. This result is also in line with the local studies taken place in various part of Ethiopia such as Dere Teyara district Eastern Ethiopia (42.8%) [28], Tehulederie district Northeast Ethiopia (44.6%) [29], Kofale District Southeast Ethiopia (41.3%) [30] and Kucha District Southern Ethiopia (44%) [31].

However, the result of this study found to be higher than studies carried out in Ruhengeri hospital Rwanda (22.3%) [32], Urban Lagos state Nigeria (31.6%) [33], East Pokot District Midwest Kenya (28%) [34], Ife central local Government Nigeria (34.9%) [35], Tharaka Nithi county Kenya (20.3%) [36], Jamnagar district India (32.2%) [37], Uttar Dinjpur District West Bengal (34.5%) [38], five hard-to-reach districts in Bangladish (24.5%) [39] and Mbarara District Uganda (35%) [40]. This figure is also higher than as compared with researches have done at different parts of Ethiopia such as Duguna Fango District South Ethiopia (18.3%) [41], Jimma Zone Southeast Ethiopia (23.3% [42], Arbaminch Zuria District Southern Ethiopia (30%) [43] and Aleta Wondo district South Ethiopia (17%) [44].

This disparity in magnitude might be secondary to variation in study settings. From these perspective, those participants who were selected from rural settings less likely have accessed for information, media and even healthcare services than those who were from urban areas. Moreover, this discrepancy might be resulted due to variation in respondent's sociodemographic characteristics. The current study finding revealed that more than 38% of respondents have attended college or university education. In contrary, other studies pointed out that only smaller number (13.4% or below), of participants could attend at college or university level of education [34, 41, 43]. Similarly, about nine-tenth of respondents in the current study were able to read and write. On the contrary, more than half number of study participants in the previous studies had never attended any formal education [34, 39, 44]. This is also true for male partners' educational status [43]. Empirical evidences pointed out that educational status has direct proportional relationship with the general health care seeking behavior and decision making power of the individuals. Furthermore, this dissimilitude might be attributed to the variation in occupation of study participants. In the current study, considerable proportion,(39%), of respondents are governmental employees whereas more than 85% of respondents in the previous studies were non-governmental employer and/or with no paid work [40, 41]. Thus, employees pose great opportunity to meet multidisciplinary professionals thereby share a number of experiences and they are likely having comprehensive awareness on health related information. In connection to this, the disparity could be explained by variation in proportion of study participants who have attended ANC visit for the indexed pregnancy. In the current study, nearly three-fifth,(58%), of the pregnant women had ANC visit unlike that of only 44% in other study [44]. Finally, this disagreement might be resulted to the variation in measurement of the outcome variables; the current study has counted those participants who mentioned, practiced or intended to practice the BP/CR components while other respondents were aiming exclusively on the practice aspect [39, 43].

On the other hand, the findings of the current study are lower as compared with a study conducted in Debre birhan town Ethiopia (65.9%) [45], Mizan-Tepi Ethiopia (66%) [19], Northern Ghana (74.3%) [46], Federal Police Referral Hospital Ethiopia (53.3%)[23], Chamwino district Central Tanzania (58.2%) [47], Edo state Nigeria (87%) [48], Nepal (65%) [49], Imo state Nigeria (77%) [50], Migori county Kenya(56%) [51], Kericho county Kenya(70.5%) [52], Chiro zonal Hospital East Ethiopia (56.7%) [53], Urban Anambra state Nigeria (54.5%) [54], Kamineni Hyderabad (71.5%) [55], and Cross River State Southeastern Nigeria (70.6%) [32].

This difference might be explained by variation in the study design since some of the previous studies were institutional based [32, 51–55]. Furthermore, the difference in magnitude could be accredited to dissimilarity in number of ANC attendants. This is true even among community based studies since more than 95% study participants in the previous studies have had ANC follow-up [47, 49, 50] while the number of respondents were only 58% in the current study at the time of interview. Hence, ANC attendees are more likely to have better awareness as well as access for knowledge and advice on BP/CR from health care providers.

In the current study, intended pregnancy had significant association with good knowledge on BP/CR. This finding was also supported by local studies conducted at Debre birhan [45] and Jimma Zone [42]. From the above results, we can deduce that intended pregnancies have enormous advantages on accessing the information, care, and follow-up services for the pregnancy. This could be due to the fact that mothers having intended pregnancy more likely seek health related information by either reading different magazines, asking friends/relatives, attending health programs from media, or attending the recommended ANC follow-up at health facilities where they could get enough information about BP/CR than their counterparts.

Women's occupation was a significant and independent factor of knowledge on BP/CR. In this study, those government employees had higher probability of having good knowledge on BP/CR than housewives. This finding was also in agreement with studies conducted in Pune City India [56], Goba district Ethiopia [57], Migori county Kenya [51], and Tharaka Nithi county Kenya [36]. This could be due to the fact that government employees are more educated and have access for different media and information sources than the general population.

ANC visit for the current pregnancy was significantly associated with knowledge on BP/CR. Those pregnant women who had ANC follow-up for their current pregnancy had better knowledge on BP/CR than a woman who did not start ANC follow-up for the current pregnancy. This finding was in accordance with the study done in Debre Birhan Ethiopia [45], Bahir Dar Ethiopia [58], Duguna Fango district South Ethiopia [41], Chamwino District Central Tanzania [47], Indore City India [25], Aleta Wondo District South Ethiopia [44], and Lekhnath Municipality Nepal [59]. This could be due to the fact that women who had ANC follow-up had greater chance of getting health professional's advice and education on BP/CR as part of the ANC service than a woman who did not attend ANC.

Being parous was found to be independent predictor of good knowledge on BP/CR that those women with history of childbirth had higher chance of having good knowledge on BP/CR than nulliparous women. This finding was also consistent with studies conducted in sub-Saharan Africa Austere [60] and Goba district Ethiopia [57]. This might be due to the fact that woman who had history of previous child birth may have better anticipations of the pregnancy related complications than a woman with no child birth experience.

## 5. Limitations

The magnitude of good knowledge on BP/CR might be underestimated since mothers' knowledge status on BP/CR could be increased throughout the pregnancy period. Hence, those mothers who had poor knowledge at time of interview might have good knowledge at the end of the pregnancy.

## 6. Conclusion

In this study, proportion of pregnant women having good knowledge on BP/CR was found to be low. Having intended pregnancy, having history of previous childbirth, being governmental employee and having ANC visit for the current pregnancy were factors which associated independently with good knowledge on BP/CR among pregnant women. Hence, stakeholders would better design strategies that encourage women to have planned pregnancy, to attend ANC visit during pregnancy, and to engage in governmental work which in turn may enhance women's knowledge on BP/CR.

## Figures and Tables

**Table 1:** Socio demographic related characteristics of pregnant women in Debre Markos town, 2017 (N=436).

**Characteristics **	**Frequency**	**Percent **
**Mother's age**		
18-24	325	74.54
>24	111	25.46
**Mother Ethnicity**		
Amhara	427	97.7
Tigre	4	0.9
Oromo	5	1.1
**Mother education**		
Unable to read & write	49	11.2
Can read &write via informal education	38	8.7
Primary	81	18.6
Secondary	99	22.7
College & above	169	38.8
**Mother occupation**		
Civil servant	170	39
Private employee	74	17
Daily labored	34	7.8
House wife	158	36.2
**Husband education **		
Un able to read & write	19	4.4
Can read to &write	21	4.8
Primary school	47	10.8
Secondary	87	20
College & above	262	60
**Husbands occupation **		
Civil servant	229	52
Private	141	32.3
Daily labored	66	15
**Family Monthly income (in US dollar)**		
< 36	45	10.3
36-90	53	12.2
>90	338	77.5

**Table 2:** Obstetric characteristics of pregnant women in Debremarkos town, 2017.

**Characteristics**	**Number**	**Percent**
**Age at 1st marriage **		
<18	30	6.9
18-24	295	67.7
>24	111	25.5
**Age at 1st pregnancy**		
<18	9	2.1
18-24	226	51.5
>24	201	46.1
**Gravidity**		
1	200	45.9
2-4	223	51.1
≥5	13	3
**Parity **		
0	198	45.4
1	136	31.2
2-4	77	17.7
≥5	25	5.7
**Desired Number of children in life **		
1-2	39	8.9
3-4	347	79.6
5-6	50	11.5
**ANC visit **		
Yes	253	58
No	183	42
**Place of ANC visit (n=253)**		
Hospital	59	23.3
Health center	194	76.7

**Table 3:** Danger signs of antepartum, intrapartum, postpartum, and new born mentioned by pregnant mothers in Debrmarkos town, Northwest Ethiopia, 2017.

**Variables**	Number	Percent
**Elements of danger sign during pregnancy**		
Vaginal bleeding	382	87.6
Severe headache	305	70
Blurred vision	197	45.2
Reduced /absent fetal movement	59	13.5

**Knowledge on danger sign during labor and childbirth**		
Excessive vaginal bleeding	362	83
Severe headache	249	57.1
Convulsion	205	47
Unconsciousness	85	19.5
Prolonged labor	75	17.2
Retained placenta	27	6.2

**Knowledge on danger sign during postpartum period **		
Severe headache	292	67
High fever	154	35.3
Foul smelling vaginal discharge	36	8.3

**Knowledge on newborn danger sign **		
Very low birth weight	210	48.2
Convulsion	196	45
Lethargy /unconsciousness	177	40.6
Breathing difficulty	175	40.1

**Table 4:** Bivariate and Multivariable logistic regression analysis for factors associated with knowledge on BP/CR among pregnant women at Debre Markos town, Northwest Ethiopia, 2017(n=436).

**Variables **	**Knowledge on BP/CR**		**COR(95**%**CI)**	**AOR(95**%**CI)**
	**Good**	**Poor**		
**Mother's age**				
18-24	202	**123**	1.21(0.42,3.76)	1.13(0.21,4.26)
>24	64	**47**	1	1
**Mothers educational status**				
Able to read &write	181	206	1.81(3.05,9.31)	1.32(0.52,2.21)
Unable to read &write	16	33	1	1
**Mothers occupation**				
Government	95	75	2.50(1.60, 3.93)	**6.50(2.5,16.87)** **∗** **∗**
Private employee	35	39	1.78(1.01,3.12)	1.5(0.79,2.87)
Daily labor	14	20	1.39(0.65,2.96)	1.4(0.60,3.2)
House wife	53	105	1	1
**Husband education**				
Can read to &write	217	200	1.21(0.42,12.71)	1.15(0.32,9.46)
Un able to read & write	9	10	1	1
**Husbands occupation**				
Civil servant	128	101	1.12(0.53,1.82)	1.05(0.36,1.49)
Private	74	67	0.98(0.28,3.74)	0.74(0.17,3.64)
Daily labored	35	31	1	1
**Monthly income (US Dollar)**				
<39	14	31	1	1
39.00-90.00	18	35	1.14(0.49, 2.66)	1.18(0.48,0.93)
>90.00	165	173	2.11(1.08,4.11)	1.54(0.75,3.20)
**Intended Pregnancy**				
Yes	177	190	2.28(1.30,3.99)	**2.13(1.16,3.90)** **∗**
No	20	49	1	1
**Parity**				
One or more	118	120	1.48(2.61,9.63)	**2.17(1.18,4.0)** **∗**
Nulipara	79	119	1	1
**ANC follow up for current pregnancy**				
Yes	190	205	4.5(1.95,10.4)	**5.50(2.20,13.70)** **∗** **∗**
No	7	34	1	1
**Gravidity**				
Primigravida	102	98	1.10(0.83,6.48)	1.04(0.72,2.47)
Multigravida	115	121	1	1
**Place of ANC visit(n=253)**				
Hospital	36	23	1.41(0.73,1.38)	1.37(0.69,1.25)
Health center	102	92	1	1
**Desired number of children**				
1-4	202	184	1.19(0.63,3.71)	1.13(0.57,2.18)
5 or above	24	26	1	1

*∗*=P-value <0.05, *∗∗*=P-value <0.01, and 1= reference category.

## Data Availability

The data sets collected and analyzed for the current study is available from the corresponding author and can be obtained on a reasonable request.

## Abbreviations

ANC: Antenatal care
AOR: Adjusted odds ratio
BP/CR: Birth preparedness and complication readiness
COR: Crude odds ratio
MMR: Maternal mortality ratio
SDG: Sustainable development goal
SSA: Sub-Saharan Africa
WCA: West & Central Africa
WHO: World Health Organization
HEWs: Health Extension Workers.
